# Blood Coagulation Parameters and Platelet Indices: Changes in Normal and Preeclamptic Pregnancies and Predictive Values for Preeclampsia

**DOI:** 10.1371/journal.pone.0114488

**Published:** 2014-12-02

**Authors:** Lei Han, Xiaojie Liu, Hongmei Li, Jiaqun Zou, Zhiling Yang, Jian Han, Wei Huang, Lili Yu, Yingru Zheng, Li Li

**Affiliations:** 1 Department of Obstetrics and Gynecology, Research Institute of Surgery, Daping Hospital, Third Military Medical University, Chongqing, P.R.China; 2 Department of Obstetrics and Gynecology, the 306th Hospital of the Chinese People’s Liberation Army, Beijing, P.R.China; 3 Department of General Surgery, Research Institute of Surgery, Daping Hospital, Third Military Medical University, Chongqing, P.R.China; University Hospital Basel, Switzerland

## Abstract

**Background:**

Preeclampsia (PE) is an obstetric disorder with high morbidity and mortality rates but without clear pathogeny. The dysfunction of the blood coagulation-fibrinolysis system is a salient characteristic of PE that varies in severity, and necessitates different treatments. Therefore, it is necessary to find suitable predictors for the onset and severity of PE.

**Objectives:**

We aimed to evaluate blood coagulation parameters and platelet indices as potential predictors for the onset and severity of PE.

**Methods:**

Blood samples from 3 groups of subjects, normal pregnant women (n = 79), mild preeclampsia (mPE) (n = 53) and severe preeclampsia (sPE) (n = 42), were collected during early and late pregnancy. The levels of coagulative parameters and platelet indices were measured and compared among the groups. The receiver-operating characteristic (ROC) curves of these indices were generated, and the area under the curve (AUC) was calculated. The predictive values of the selected potential parameters were examined in binary regression analysis.

**Results:**

During late pregnancy in the normal pregnancy group, the activated partial thromboplastin time (APTT), prothrombin time (PT), thrombin time (TT) and platelet count decreased, while the fibrinogen level and mean platelet volume (MPV) increased compared to early pregnancy (*p*<0.05). However, the PE patients presented with increased APTT, TT, MPV and D-dimer (DD) during the third trimester. In the analysis of subjects with and without PE, TT showed the largest AUC (0.743) and high predictive value. In PE patients with different severities, MPV showed the largest AUC (0.671) and ideal predictive efficiency.

**Conclusion:**

Normal pregnancy causes a maternal physiological hypercoagulable state in late pregnancy. PE may trigger complex disorders in the endogenous coagulative pathways and consume platelets and FIB, subsequently activating thrombopoiesis and fibrinolysis. Thrombin time and MPV may serve as early monitoring markers for the onset and severity of PE, respectively.

## Introduction

Preeclampsia (PE) is an intractable obstetric disorder that affects 6–8% of pregnancies worldwide. PE is characterized by hypertension (blood pressure ≧140/90 mmHg), proteinuria (≧0.3 g/d), edema and other symptoms and may begin as early as the 20th gestational week and last for 6 weeks after delivery. Furthermore, PE has high morbidity and mortality rates [1]. The pathogenesis of PE remains unknown, and the many theories related to the etiology of PE pose great challenges for future investigation. The abnormal invasion of placenta and the release of placenta-derived adverse factors during the first trimester is thought to be the main cause of the extensive damage to the maternal endothelium and systemic inflammatory response involving many systems and organs in late pregnancy [2]. To date, there is no effective treatment for PE in addition to the termination of pregnancy. Therefore, a reliable predictor for PE would play an important role in early prevention and intervention. PE can be classified into two degrees, mild PE (mPE) and severe PE (sPE), and there are different treatments and clinical outcomes for each degree [3]. It is necessary to predict the severity of PE for rational gestational management [4].

In PE patients, the coagulation-fibrinolytic system is thought to be one of the most seriously affected systems by maternal inflammatory reactions and immune dysfunction [5]. The balance between coagulation and anticoagulation is vital to the regulation of utero-placental circulation and organ perfusion in pregnant woman. An appropriate increase in blood coagulation is important for normal pregnant woman to reduce postpartum hemorrhage and to limit other complications [6]. When this balance is upset in PE patients, the bloodstream of the placenta and many organs is blocked by microthrombosis [7]. The super-hypercoagulable state of women with PE may also lead to systematic disorders of metabolism as well as multiple organ dysfunction and may even threaten maternal and fetal lives. Therefore, coagulative and fibrinolytic status is a good predictor for the onset and clinical degree of PE. In recent years, studies with a varying number of subjects have shown that many indices, such as D-dimer (DD) [8], soluble vascular endothelial growth factor receptor (sFlt) [9] and platelet distribution width (PDW) [10] are ideal indicators for PE. However, there are many conflicting conclusions because of the diverse effects on the coagulation-fibrinolytic system in late pregnancy of patients with PE. Furthermore, the involvement of patients with hemolysis, elevated liver enzymes and low platelet count (HELLP) syndrome, immune thrombocytopenic purpura (ITP) or gestational thrombocytopenia (GT) would necessarily cause a confounding bias [11]. Therefore, we aimed to eliminate these interfering factors to investigate the variation in laboratory markers of the coagulation-fibrinolytic system and to determine their potential value in predicting the onset and severity of PE for early intervention.

## Subjects and Methods

This retrospective case-control study enrolled 173 pregnant women: 53 women who were diagnosed with mPE, 41 with sPE and 79 normal pregnancies. All pregnancies were monocyesis and were delivered between January 2011 to December 2013 in the Department of Obstetrics and Gynecology, Daping Hospital, the 3^rd^ Military Medical University. The gestational stage of each subject was verified by ultrasonography using the fetal crown-rump length during the 10^th^ to 12^th^ week of gestation.

### Inclusion criteria

Patients with either mPE or sPE were diagnosed according to the criteria of the ACOG Practice Bulletin [12]. Mild PE was defined as new onset of blood pressure ≧140/90 mmHg on more than two readings taken 6 h apart after 20 weeks gestation, combined with proteinuria ≧0.3 g/24 h, but not meeting the standards for sPE. Severe PE was defined as blood pressure ≧160/110 mmHg, serious proteinuria (≧2 g/24 h) and manifestations of multiple organ damage or dysfunction, for example, headache, pulmonary edema, oliguria or even fetal growth restriction (FGR). However, women with pre-existing renal disease, insulin-dependent diabetes, asthma requiring steroidal treatment, chronic hepatitis (with or without hepatic dysfunction), severe trauma history, anticoagulant drug-use history, oral contraceptive-use history, smoking history, ITP, HELLP syndrome, GT or any hematological diseases were all excluded. Normal pregnancies were all healthy women with a singleton gestation and a normal obstetric history. Blood pressures of the women in this group were less than 140/90 mmHg, and these patients had no proteinuria. Pregnant women in the normal group all delivered full-term congenitally healthy live births. Written informed consent was received from all participating patients and their families, who agreed that their results of laboratory tests could be used for additional research. The whole design, procedure and informed consent of this study were approved by the Research Ethics Committee of Daping Hospital.

### Blood collection and laboratory methods

Blood samples were all fasting blood and were drawn by a qualified phlebotomist using a 19-G needle. A 2-mL blood sample from each patient was collected into a vacuum tube (purple cap) containing 2.0 mg/mL EDTA-2K and preserved at 37°C for platelet analysis. The blood sample was measured using the LH 755 automatic quantitative hematology analyzer (BECKMAN COULTER Inc., U.S.A.). The blood sample for coagulative function factors was collected into a vacuum tube (blue cap) containing sodium citrate (32.06 mg/mL, final concentration 3.8%) in a 9∶1 volume ratio. Platelet poor plasma (PPP) was prepared by double centrifugation at 2500 *g* for 15 minutes with supernatant separation and was divided into a quadrisection. One part of the blood plasma was kept at room temperature for DD testing, and the remainder was frozen at −80°C until required. The samples were analyzed using the ACL-TOP700 automatic blood coagulation analyzer (BECKMAN COULTER Inc., U.S.A.). The Human specific Imu-clone enzyme linked immunosorbent assay (ELISA) Kit (American Diagnostica Inc., U.S.A.) was used for DD testing following the manufacturer’s instructions. All tubes were mixed by inverting the tubes 5–10 times immediately after the blood draw and were sent to the clinical laboratory of Daping Hospital for analysis within 1 h.

### Statistical analysis

The variables are expressed as the mean ± standard deviation (SD). The statistical significance of parametric variables among the different groups was performed using multiple comparisons performed by one-way analysis of variance (ANOVA) and non-parametric variables by the Kruskal-Wallis test with post-hoc analysis. Pairwise comparisons were applied to compare the same index of one subject at early and late gestational stages, either by Fisher’s LSD for parametric variables or by the Wilcoxon test for non-parametric variables. All tests were 2-sided with 95% confidence intervals (95% CIs). The receiver-operating characteristic (ROC) curves were generated to estimate the utility of each parameter as a tool for predicting the severity of PE using the Statistical Package for Social Sciences version 18.0 (SPSS Inc., U.S.A.). The area under the ROC curves and the related parameters were also calculated in order to analyze the diagnostic points. The predictive value of the indicators was examined through the binary regression analysis at last. Statistical significances were all determined on the basis of *p*<0.05.

## Results

### Subject characteristics

All pregnant women in this study were nulliparous and had regular prenatal care by a physician in the Department of Obstetrics and Gynecology, Daping Hospital. Demographic and clinical characteristics of the participants are presented in Table 1. The difference in age and body mass index (BMI) at term among the three groups (normal, mPE and sPE) showed no statistical significance (Table 1, *p*>0.05). There were also no significant differences in the gestational dates of testing for platelet and coagulation-fibrinolytic indices during both early and late pregnancy among the three groups (Table 1, *p*>0.05). These fundamental indices ensured the homogeneity of the clinical samples. As expected, both mPE and sPE patients had significantly higher systolic and diastolic blood pressure than healthy pregnant women (Table 1, *p*<0.05). Patients with mPE and sPE showed vastly increased 24-h urine protein (1.1±0.6 g and 3.9±1.5 g, respectively) compared to zero urine protein found in the normal pregnant group (Table 1, *p*<0.05). The pregnancy duration of the sPE group (246.4±3.1 d) was significantly shorter than that of the normal pregnancy group (266.1±4.6 d), and the mPE and sPE groups had lighter birth weights (2.8±0.4 kg and 2.6±0.4 kg, respectively) than that of the normal group (3.3±0.2 kg) (Table 1, *p*<0.05). These two differences reflected the maternal and perinatal adverse outcomes resulting from PE.

**Table 1:** Maternal demographic and clinical characteristics.

	Normal pregnancy (n = 79)	Preeclamptic pregnancy		*p*-Value
		mPE (n = 53)	sPE (n = 41)	
Age (y)	26.7±2.3	27.2±1.9	27.5±1.6	0.87
BMI at term (kg/m^∧^2)	23.4±1.2	23.8±0.8	22.3±0.9	0.34
Early pregnant test day (d)	81.7±3.5	80.5±3.2	78.4±2.8	0.53
Late pregnant test day (d)	250.5±2.8	242.3±2.4	241.7±3.3	0.61
Systolic blood pressure (mmHg)	115.6±7.8	151.4±5.5*	169.2±5.7*	<0.01
Diastolic blood pressure (mmHg)	78.2±4.9	98.4±6.3*	119.8±5.2*	<0.01
24 h urine protein (g)	0.0±0.0	1.1±0.6*	3.9±1.5*	<0.01
Pregnancy duration (d)	266.1±4.6	257.6±3.7	246.4±3.1*	0.03
Birth weight (kg)	3.3±0.2	2.8±0.4*	2.6±0.4*	<0.01

Note: * compared with normal pregnancy group, *p*<0.05.

### Changes in blood coagulation parameters and platelet indices during the early and late pregnant stages

In these subjects, the level of activated partial thromboplastin time (APTT) significantly decreased from 29.0±2.5 s in early pregnancy to 27.7±2.4 s in late pregnancy (*p*<0.05), and prothrombin time (PT) also shortened remarkably from 9.9±0.5 s to 9.6±0.6 s (*p*<0.05). However, the plasma level of fibrinogen (FIB) and thrombin time (TT) did not change significantly as gestational stage progressed (*p*>0.05).

In the third trimester, the platelet count (Pt Count) of these pregnant women sharply reduced to 164.9±51.1 10^9^/L from 185.7±47.2 10^9^/L in the first trimester (*p*<0.05), but their mean platelet volume (MPV) significantly elevated from 9.5±1.1 fL during early pregnancy to 10.4±1.4 fL during late pregnancy (*p*<0.05). Nevertheless, there were no significant differences in PDW or platelet ratio (Pt Ratio) between the first and the third trimesters (*p*>0.05).

**Figure 1:**
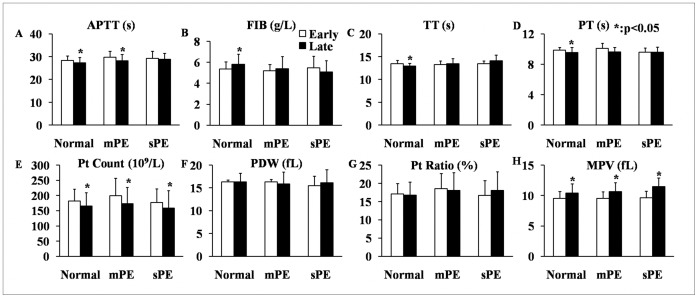
Pairwise comparisons of blood coagulation parameters and platelet indices of healthy pregnant women (Normal group), mPE patients (mPE group) and sPE patients (sPE group) between early and late pregnancy.

Further analysis of the characteristics of these coagulative parameters and platelet indices showed that APTT, PT and TT in normal pregnant women all significantly decreased, while FIB increased during the late gestational weeks compared to early pregnancy (Figure 1A, B, C, D, *p*<0.05). The level of APTT also decreased notably during the third trimester compared to the first trimester (Figure 1A, *p*<0.05). However, the APTT of the sPE patients and the remaining three coagulative parameters of both the mPE and sPE patients did not vary greatly (Figure 1A, B, C, D, *p*>0.05). The platelet count and MPV of all three subject groups rose as gestational weeks progressed to late pregnancy; these results were consistent with the results of the normal pregnant subjects (Figure 1E,H, *p*<0.05). The PDW and Pt Ratio of all subjects during late pregnancy were consistent with the levels of those measures observed during early pregnancy, irrespective of the patients’ PE status (Figure 1F,G, *p*>0.05).

### Differences in blood coagulation parameters and platelet indices between healthy pregnant women, mPE patients and sPE patients during late pregnancy

**Figure 2:**
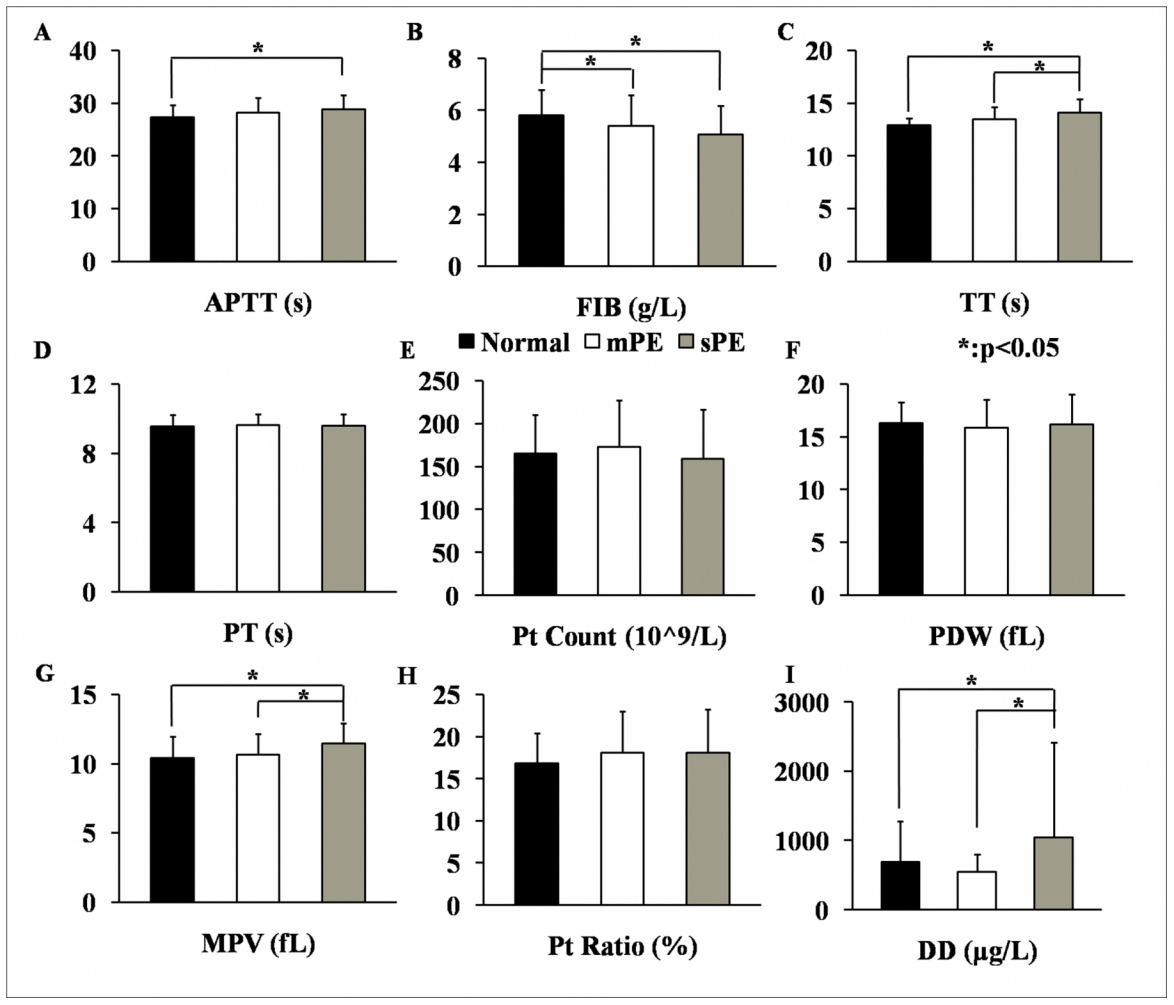
Comparisons of blood coagulation parameters and platelet indices in healthy pregnant women (Normal group), mPE patients (mPE group) and sPE patients (sPE group) during the late pregnant stage.

At the late pregnancy stage, the levels of APTT and TT of the sPE patients were 28.9±2.7 s and 14.1±1.3 s, respectively, which were significantly higher than those of the healthy pregnant women (27.3±2.4 s and 12.9±0.6 s, respectively) (Figure 2A and C, *p*<0.05). In addition, the TT of the sPE patients was higher than that of the mPE patients (13.5±1.2 s) (Figure 2C, *p*<0.05). The FIB of the normal group during the third trimester was 5.8±1.0 g/L, which was remarkably higher than that for either the mPE patients (5.4±1.2 g/L) or the sPE patients (5.1±1.1 g/L) (Figure 2B, *p*<0.05). Among the four platelet indices, MPV was the only index showing statistical significance among the different groups in late pregnancy (Figure 2E, F, G, H): 11.4±1.4 fL in the sPE group compared to 10.6±1.5 fL in the mPE group and 10.4±1.6 fL in the normal group (Figure 2G, *p*<0.05). The concentration of DD was routinely measured during the third trimester in our hospital. The concentration of DD in the healthy pregnant woman was 687.1±592.5 µg/L and was 542.1±259.3 µg/L in the mPE group. However, the concentration of DD soared to 1037.7±1381.8 µg/L in the sPE group, which was significantly higher than that in the other two groups (Figure 2I, *p*<0.05).

### Analysis of ROC curves for blood coagulation parameters and platelet indices for predicting the onset of PE

**Figure 3:**
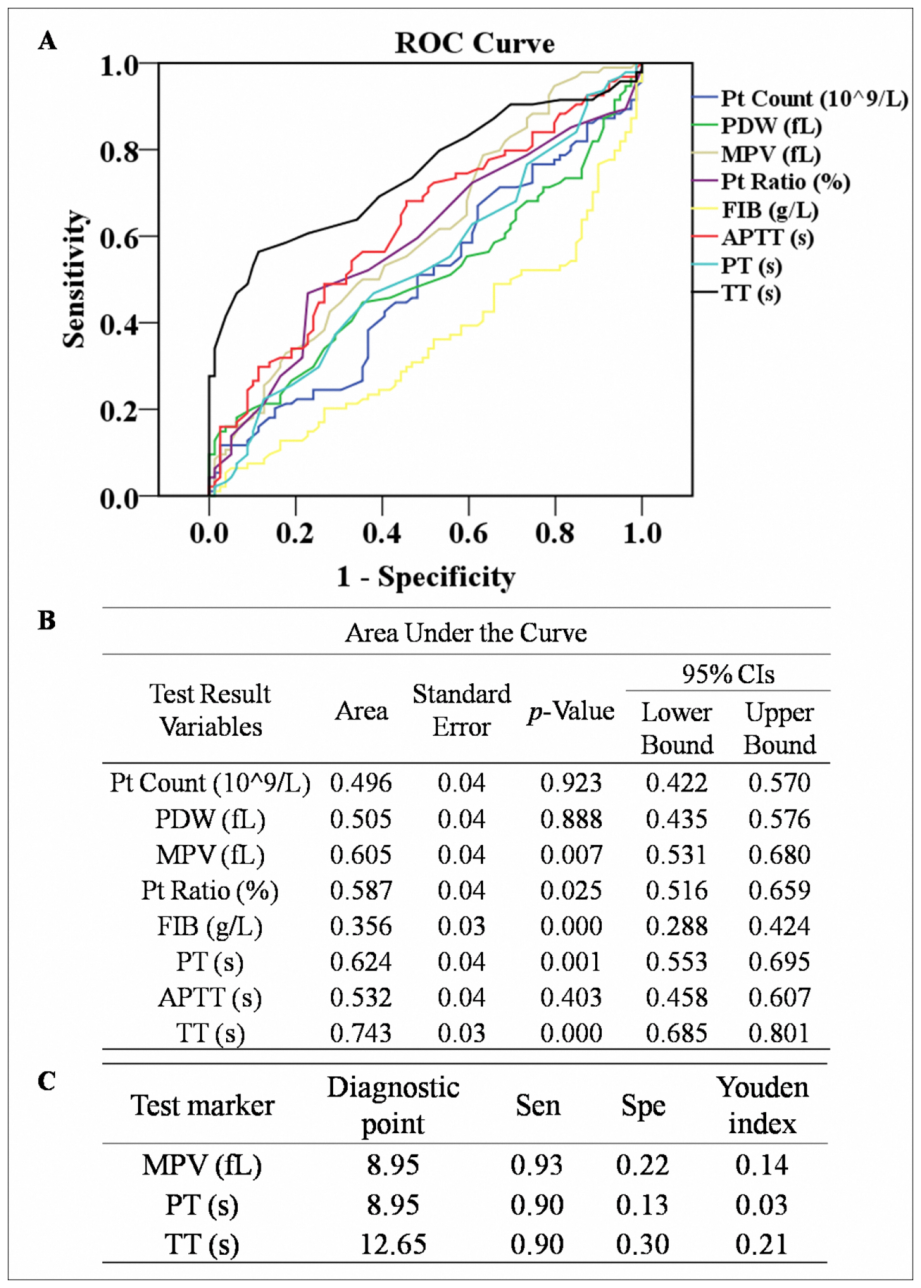
ROC curves for APTT, FIB, TT, PT, Pt Count, PDW, MPV and Pt Ratio in early pregnancy to analyze the optimal diagnostic points for predicting preeclampsia in women.

The ROC curves for APTT, FIB, TT, PT, Pt Count, PDW, MPV and Pt Ratio for the subjects in early pregnancy are presented in Figure 3A. MPV, FIB, PT and TT changed significantly between patients with PE and those with a healthy pregnancy (*p* = 0.007, *p*<0.001, *p* = 0.001 and *p*<0.001, respectively); of the eight parameters, these were considered to be possible predictors of PE (Figure 3B). The areas under the curve (AUC) of MPV and PT were 0.605 (95% CI of 0.531–0.680) and 0.624 (95% CI of 0.553–0.695), respectively (Figure 3B), which indicated that these parameters could be used as indicators for PE. However, the AUC of TT was 0.743, making it ideal for predicting PE in the subjects analyzed. The exact diagnostic points of TT, MPV and PT were 12.65 s, 8.95 fL and 8.95 s, respectively, when the individual sensitivity (Sen) of these indices was only just above 0.90 (0.90, 0.93 and 0.90, respectively). Meanwhile, their specificities (Spe) were 0.30, 0.22 and 0.13, and their Youden index values were 0.21, 0.14 and 0.13, respectively (Figure 3C).

### Analysis of ROC curves for blood coagulation parameters and platelet indices for predicting the severity of PE

**Figure 4:**
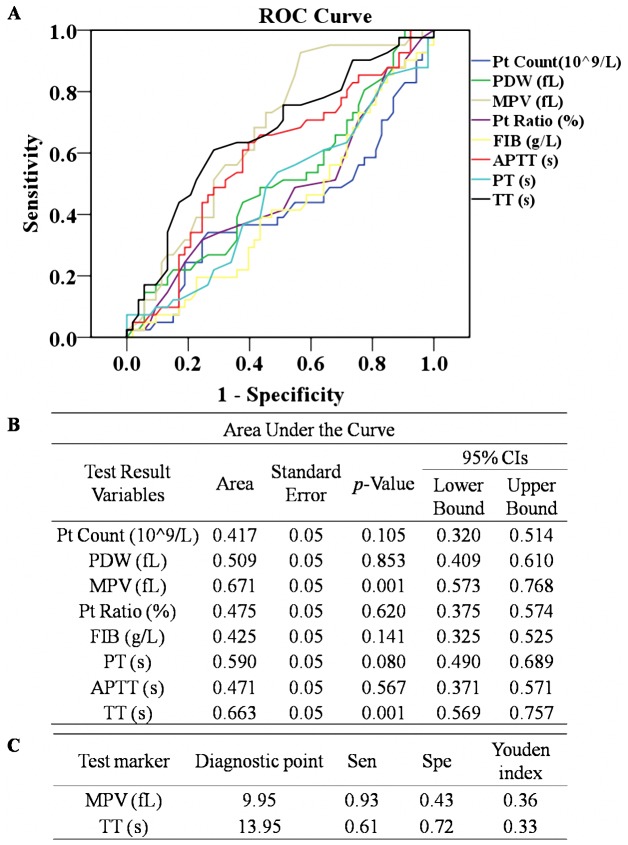
ROC curves for APTT, FIB, TT, PT, Pt Count, PDW, MPV and Pt Ratio in early pregnancy to analyze the optimal diagnostic points for predicting the severity of PE.

Figure 4A shows the ROC curves for the eight blood coagulation parameters and platelet indices for patients with both mPE and sPE during the first trimester. MPV and TT both had remarkable differences in the mPE and sPE groups, with the *p*-value below 0.05 (*p* = 0.001) (Figure 4B). These two parameters presented large AUCs of 0.671 (95% CI of 0.573–0.768) and 0.663 (95% CI of 0.569–0.757) (Figure 4B), which suggests that MPV and TT are optional markers for predicting the severity of PE. At Youden index peak levels of 0.36 for MPV and 0.33 for TT, the diagnostic points of these two markers were 9.95 fL and 13.95 s, respectively (Figure 4C).

### Regression analysis of the predictive blood coagulation parameters and platelet indices

These predictive blood coagulation parameters and platelet indices selected through the analysis of ROC curves were also taken into regression analysis to descript their predictive values qualitatively. The platelet indices MPV and blood coagulation parameter TT have relatively high values to predict the onset of PE (Table 2). But MPV seemed to be more reliable in predicting the severity of PE (Table 2).

**Table 2:** Regression analysis of predictive parameters.

Predicting content	Blood coagulation parameters and platelet indices	Significance	Relative risk
Onset of PE	MPV	0.009	3.725
	PT	0.519	1.369
	TT	0.003	3.450
Severity of PE	MPV	0.001	12.797
	TT	0.371	0.676

## Discussion

The physiological pro-coagulation state, also known as hypercoagulable status, in pregnant women has important significance for human reproduction [13]. The parameters of blood coagulation function for all subjects in our study trended toward increasing coagulation; this was true for women who had both healthy pregnancies and preeclamptic pregnancies (Figure 1.A and D). The APTT and the PT of all subjects reflects the function of endogenous and exogenous coagulative pathways, respectively [14]. Interestingly, further research has shown that the differences in significance for APTT and PT are mainly attributed to changes in the normal group (Figure 1.A and D). In the mPE group, APTT had the same trend as that in the normal group (Figure 1.A), which indicated that the endogenous coagulative pathway and factors of mPE patients had not suffered significantly. However, in the sPE patients, the physiological change in the endogenous coagulative pathway from early to late pregnancy was notably changed; the average level of APTT in the sPE group during the third trimester was significantly higher than in the normal group (Figure 2.A). This result suggests that a certain degree of coagulative dysfunction occurs in the endogenous coagulative pathways of sPE patients, while their exogenous coagulative pathways do not change greatly.

Thrombin time can reflect the content and structure of plasma FIB to some degree [15]. Thrombin time and FIB usually has opposite changing trend from the first to the third trimester in pregnant women, which also happened in the healthy pregnancies from our study (Figure 1.B and C). However, there were no differences in FIB and TT between early and late pregnancies of women who developed PE, although PE is well known to be a super-hypercoagulable status combining the rise of thrombin with a rise of coagulation factors [16]. Furthermore, the concentration of FIB has been reported to increase in proportion to the severity of PE and is higher in the plasma of PE patients [17]. However, in our results, the level of FIB was lower in the sPE and mPE groups than in the normal group during the late gestational period, while conversely, the TT was higher in the sPE group than in the other two groups (Figure 2B). This result illustrates that the thrombogenic and fibrinolytic systems of PE patients undergo complex and profound changes rather than simply having an increase of procoagulant substances. On one hand, the synthesis of endogenous clotting factors and FIB in the liver may be insufficient in the late pregnancy of PE patients due to overloaded hepatic function and decreased total plasma protein [18]. On the other hand, the systemic super-hypercoagulable state and diffuse microthromboses in preeclamptic circulation may also deplete a large amount of FIB [19]. Strict criteria were imposed in this study to exclude most extraneous factors that influence coagulation and anticoagulation, such as multiple pregnancies and anticoagulant drug history [20]. Therefore, it is reasonable to believe that the endogenous coagulative pathway is abnormally injured and that FIB concentration decreases in the coagulative and fibrinolytic systems of PE patients during the third trimester. And TT may be an ideal parameter for predicting the onset of PE.

D-dimer is a specific degradative product resulting from the hydrolysis of the fibrin monomer and is considered to be an indirect marker for thrombosis and fibrinolytic activity. The maternal DD concentration in normal pregnancy increases progressively from conception to delivery [8]. Previous research and our present investigation all showed higher DD concentrations in pregnant women with PE, especially in women with sPE compared to normotensive women [16], [21]. D-dimer is involved in the dynamic balance between plasminogen activators (t-PA and uPA) and plasminogen inhibitor (PAI-1) in women with preeclampsia [22]; therefore, DD concentration can reflect the dynamic changes in both the super-hypercoagulable status and the activated fibrinolytic state in PE patients.

Blood platelets participate in the pathological processes of hemostasis and thrombosis and are regenerated from megakaryocytes in bone marrow hematopoietic tissues [10]. Therefore, platelet indices, such as Pt Count, Pt Ratio, MPV and PDW, are valuable markers for thromboembolic diseases and platelet activation [23]. The Pt Counts of the subjects in our study decreased in late pregnancy compared to early pregnancy (Figure 1.E), and this trend was found in all three subject groups (Figure 1.E). However, the Pt Count was not significantly different among the normal, mPE and sPE groups during the third trimester, which indicates that the decreased Pt Count is mainly caused by gestation itself rather than the PE. Therefore, although Pt Count is an important clinical characteristic of PE, it should not be considered an absolute marker for the progression of PE [12]. We speculate that the reason Pt Count failed to be identified as a marker for PE in our study may be due to our elimination of patients with HELLP syndrome, GT and abnormal liver enzymes. The strict screening and good homogeneity of the subjects may also be the reason for our finding of no significant difference in PDW among the different groups and gestational periods, despite the fact that PDW has been considered a potential predictive maker for PE in previous research. The MPV of our participants increased during pregnancy (Figure 1.H) in the normal, mPE and sPE groups (Figure 1.H). Moreover, the disparities in MPV, consistent with a previous report [24], were more prominent in women with sPE (Figure 2.G). This significant difference in MPV can be attributed to both the process of pregnancy and a complication of PE, which is in agreement with results from previous studies [25]. Together, the alterations of Pt Count and MPV adequately reflect the dynamic balance between massive consumption in the peripheral blood and the continuous activation of blood platelets in the bone marrow during pregnancy. Furthermore, sPE may result in the increase of MPV regulated by thrombopoietin, which then enhances coagulation activity [11].

Previous research concerning predictors of PE has mainly focused on isolating individual factors, for example, DD and sFlt, rather than on aggregative indicators [8], [9]. However, PE is a well-known multi-organ disease with systemic dysfunctions. Therefore, it is more reasonable to establish functional indices for predicting the onset and severity of PE. These blood coagulation parameters and platelet indices require no added expenditure because they are included in the routine prenatal examination of hospitals in most developing countries and in almost all developed countries. In this study, the ROC curve analysis shows that TT has the highest predictive value for PE followed by PT and MPV (Figure 3.B). Among them, TT is a functional indicator reflecting the in vivo metabolism of the anticoagulant substance as well as fibrinogen activity [15]. As a potential early screening marker for PE in a population of pregnant women, the sensitivity of TT should be above 0.9 to minimize false negatives. Therefore, we identified a TT value of >12.65 s as the optimal diagnostic point for predicting the onset of PE (Figure 3.C). However, the laboratory results of TT are highly variable depending on the reagents and test supplier used. Thus there may be subtle difference in the diagnostic point of TT between different hospitals. In predicting the severity of PE, MPV has slightly higher efficacy than TT (Figure 4.B), which is identical to results found in previous studies [10], [24]. MPV is a more responsive indicator than Pt Count to define the variation of platelets in early pregnancy [11], [26]. In view of the adverse consequences of both false positive and false negative predictions, the diagnostic points of MPV with the highest Youden Index is selected as the optimal cut-off level: >9.95 fL (Figure 4.C). In addition, the predicting efficiency of MPV for the severity of PE is also desirable (Table 2).

However, as a retrospective study, the integrity and homogeneity of above-mentioned cases are limited. It is difficult to look for a proper control group of patients, and to ensure the standardization and consistency of clinical laboratory tests. The polycentric investigations that involve massive data with high level of clinical value and reliability are main object of our clinical research work in the future.

## Conclusion

Normal late pregnancy shows a physiological hypercoagulable state with decreased levels of APTT, PT, and TT and an increased level of FIB compared to early pregnancy. This result may be caused by platelet consumption and aggregation followed by a secondary regeneration. However, the onset of PE, in particular sPE, mainly results in complex disorders in the endogenous but not exogenous coagulative pathway, which results in the consumption of platelets and FIB, with subsequent feedback activation of thrombopoiesis and fibrinolysis. Therefore, in the third trimester, PE patients present with a super-hypercoagulable state along with the prolongation of APTT and TT, the augmentation of DD and the enlargement of MPV. Among the blood coagulation parameters and platelet indices, TT is considered to be an ideal early monitoring index for PE, with an optimal diagnostic value of >12.65 s, while MPV (with optimal cut-off level of >9.95 fL) is a potential marker for predicting the severity of PE in early pregnancy.
